# Is there an association between cutaneous leishmaniasis and skin cancer? A systematic review

**DOI:** 10.12688/wellcomeopenres.15367.1

**Published:** 2019-07-23

**Authors:** Rodrigo M. Carrillo-Larco, J. Gonzalo Acevedo-Rodriguez, Carlos Altez-Fernandez, Karol Ortiz-Acha, Cesar Ugarte-Gil

**Affiliations:** 1 Department of Epidemiology and Biostatistics, School of Public Health, Imperial College London, London, UK; 2 CRONICAS Centre of Excellence in Chronic Diseases, Universidad Peruana Cayetano Heredia, Lima, Peru; 3 Centro de Estudios de Poblacion, Universidad Catolica los Ángeles de Chimbote (ULADECH-Catolica), Chimbote, Peru; 4 Instituto de Medicina Tropical Alexander von Humboldt, Universidad Peruana Cayetano Heredia, Lima, Peru; 5 Facultad de Medicina Alberto Hurtado, Universidad Peruana Cayetano Heredia, Lima, Peru; 6 Department of Clinical Research, London School of Hygiene and Tropical Medicine, London, UK; 7 Department of International Health, Johns Hopkins Bloomberg School of Public Health, Baltimore, USA

**Keywords:** Systematic review, neglected tropical diseases, tropical medicine, neoplasms, risk factors, non-communicable diseases, multi-morbidity, syndemics

## Abstract

**Background:** Cutaneous leishmaniasis is a prevalent communicable disease in low- and middle-income countries, where non-communicable diseases like skin cancer are on the rise. However, the study of multi-morbidity or co-morbidity between communicable and non-communicable diseases is limited, and even null for some tropical or neglected diseases. Nevertheless, looking at these conditions together instead of as isolated entities in places where these illnesses exist, could show new prevention and treatment paths. We aimed to summarize and critically appraise the epidemiological evidence on the association between cutaneous leishmaniasis and skin cancer.

**Methods:** Following the PRISMA guidelines, we conducted a systematic review using five search engines (Embase, Medline, Global Health, Scopus and Web of Science). We sought observational studies in which the outcome was skin cancer whilst the exposure was cutaneous leishmaniasis; these conditions should have had laboratory or pathology confirmation.

**Results:** No epidemiological investigations have studied the association between cutaneous leishmaniasis and skin cancer. Most of the evidence about the association of interest is still based on case reports and other clinical observations rather than strong epidemiological observational studies.

**Conclusions:** Research is much needed to verify the repeatedly clinical observation that cutaneous leishmaniasis may be a risk factor for skin cancer. This evidence could inform and guide early diagnosis or prevention of skin cancer in survivors of cutaneous leishmaniasis or where cutaneous leishmaniasis is still highly prevalent.

**Registration:** PROSPERO ID CRD42018111230; registered on 16/10/18.

## Introduction

Globally, non-melanoma skin cancer was among the top ten malignancies with the highest incidence in 2016 ^1^. A key driver of this high incidence is the aging of the population ^1^. Therefore, those living in low- and middle-income countries (LMICs) are at high risk, because although sanitation and health care have improved thereby delaying mortality, the preventive and treatment care for non-communicable diseases (e.g., neoplasms) is still limited.

In comparison to people in high-income countries, people in LMICs have a double burden of disease, i.e., more cases of non-communicable diseases while still facing infectious/communicable illnesses ^2^. This epidemiological profile, along with health and socio-economic inequalities, and even climate change, make it relevant to better understand infectious/communicable illnesses that may become risk factors for non-communicable diseases. In this line, cutaneous leishmaniasis (CL) have been proposed as a risk factor for skin cancer ^3– 6^.

Despite the pathophysiological background ^4, 5^, and reviews which summarized case reports ^3, 6^, to the best of our knowledge no other work has synthetized the epidemiological evidence on the association between CL and skin cancer. Aiming to provide robust epidemiological conclusions about the association of CL and skin cancer, we conducted a systematic review of observational studies. 

## Methods

### Protocol and registration

This is a systematic review of the scientific literature which protocol was registered at PROSPERO ( CRD42018111230). The work and reporting adhered to the PRISMA guidelines ^7^.

### Eligibility criteria

We sought reports that studied men and women of any age; the study sample could have been population- or hospital-based. The comparison group included people without history of CL. The outcome of interest was skin cancer, including: basal cell carcinoma, squamous cell carcinoma and epidermoid carcinoma ^3, 6^. Both the exposure and outcome of interest should have had laboratory or pathology confirmation. The eligibility criteria included observational studies with a formal comparison group, including cross-sectional, case-control and cohort studies.

### Information sources and search

The search was conducted in Embase, Medline and Global Health (these three through Ovid), Scopus and Web of Science. The search was conducted from inception to December 15 ^th^, 2018; no language restrictions were set. The search terms are available in Table 1.

**Table 1.** Search terms as used in each search engine.

Ovid, including Embase, Medline and Global Health	1	*Leishmaniasis/
	2	skin leishmaniasis/
	3	spundia.mp.
	4	leishman$.mp.
	5	cutaneous leishmaniasis.mp.
	6	(solitary or limited or old world or localised or diffuse or cutaneous).mp.
	7	5 and 6
	8	1 or 2 or 3 or 4 or 7
	9	skin cancer.mp.
	10	cutaneous malignanc$.mp.
	11	skin malignanc$.mp.
	12	skin neoplasm$.mp.
	13	(basal ADJ0 cell ADJ0 carcinoma$1) OR (basal ADJ0 cell ADJ0 neoplasm$1) OR (basal ADJ0 cell ADJ0 cancer$1)
	14	(squamous ADJ0 cell ADJ0 carcinoma$1) OR (squamous ADJ0 cell ADJ0 neoplasm$1) OR (squamous ADJ0 cell ADJ0 cancer$1)
	15	(epidermoid ADJ0 carcinoma$1) OR (epidermoid ADJ0 cell ADJ0 neoplasm$1) OR (epidermoid ADJ0 cell ADJ0 cancer$1)
	16	9 or 10 or 11 or 12 or 13 or 14 or 15
	17	8 and 16
	18	exp animals/ not humans.sh.
	19	17 not 18
	20	remove duplicates from 19
Scopus	((ALL(spundia) OR ALL(leishman*) OR ALL(Leishmaniasis) OR ALL(skin leishmaniasis)) OR (ALL(cutaneous leishmaniasis) AND (ALL(solitary) OR ALL(limited) OR ALL(old world) OR ALL(localised) OR ALL(diffuse) OR ALL(cutaneous)))) AND ((ALL(skin cancer) OR ALL(cutaneous malignanc*) OR ALL(skin malignanc*) OR ALL(skin neoplasm*)) OR (ALL((basal W/0 cell W/0 carcinoma*) OR (basal W/0 cell W/0 neoplasm*) OR (basal W/0 cell W/0 cancer*))) OR (ALL((squamous W/0 cell W/0 carcinoma*) OR (squamous W/0 cell W/0 neoplasm*) OR (squamous W/0 cell W/0 cancer*))) OR (ALL((epidermoid W/0 carcinoma*) OR (epidermoid W/0 cell W/0 neoplasm*) OR (epidermoid W/0 cell W/0 cancer*)))) NOT DBCOLL(medl) AND (LIMIT-TO ( DOCTYPE , “ar”)) AND ( LIMIT-TO(SUBJAREA, “MEDI”))	
Web of Science	((TS=(spundia) OR TS=(leishman*) OR TS=(Leishmaniasis) OR TS=(skin leishmaniasis)) OR (TS=(cutaneous leishmaniasis) AND (TS=(solitary) OR TS=(limited) OR TS=(old world) OR TS=(localised) OR TS=(diffuse) OR TS=(cutaneous)))) AND ((TS=(skin cancer) OR TS=(cutaneous malignanc*) OR TS=(skin malignanc*) OR TS=(skin neoplasm*)) OR (TS=((basal W/0 cell W/0 carcinoma*) OR (basal W/0 cell W/0 neoplasm*) OR (basal W/0 cell W/0 cancer*))) OR (TS=((squamous W/0 cell W/0 carcinoma*) OR (squamous W/0 cell W/0 neoplasm*) OR (squamous W/0 cell W/0 cancer*))) OR (TS=((epidermoid W/0 carcinoma*) OR (epidermoid W/0 cell W/0 neoplasm*) OR (epidermoid W/0 cell W/0 cancer*)))) AND DOCUMENT TYPES: (Article)	

### Study selection

Following the selection criteria above described, two independent reviewers screened titles and abstracts (R.M.C-.L., J.G.A.-R., C.A-.F. and K.O-.A., working in pairs); discrepancies were solved by consensus among the reviewers. The full-text of the selected reports was studied in detail by two reviewers independently (R.M.C-.L., J.G.A-.R., C.A-.F. and K.O-.A., working in pairs); again, discrepancies were solved by consensus among the reviewers.

### Ethics

This study was classified as of low risk because no human subject was studied. This is a systematic review of the scientific literature, which is public and can be accessed.

## Results

### Study selection

**Figure 1.**
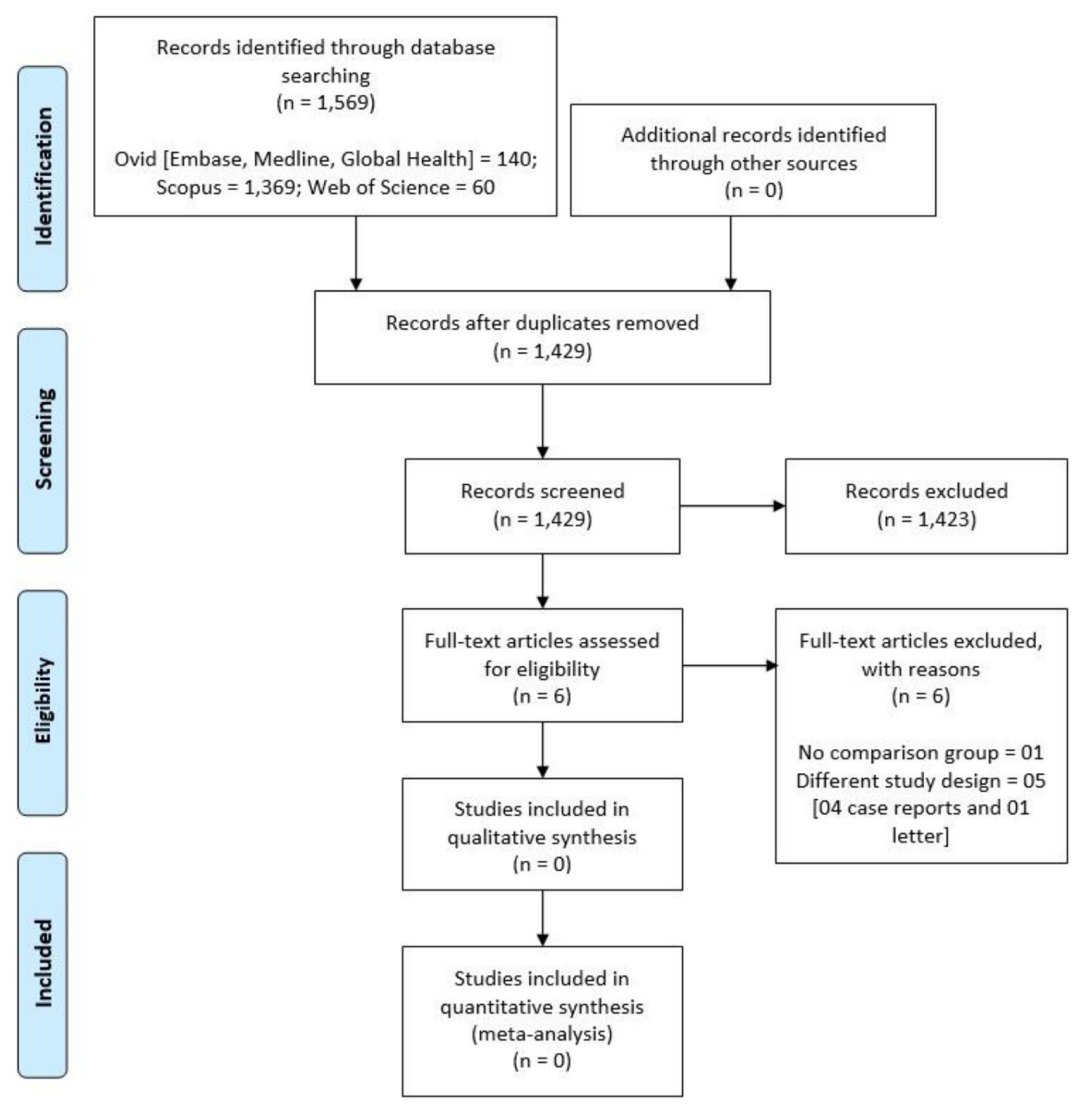
Flowchart of the review process.

As presented in Figure 1, 1,429 titles and abstracts were screened, and 6 reports were studied in detail. No reports met our selection criteria, with most of the studies being case reports or letters about the association of interest without presenting results following an epidemiological study design. Therefore, zero observational epidemiological studies have aimed to assess or quantify the association between CL and skin cancer.

## Discussion

### Summary of evidence

We conducted a thorough systematic review including relevant terms and several search engines, though this work could not find any epidemiological evidence on the association between CL and skin neoplasms. Although there were many case reports signalling how seemingly evident this association is in clinical practice ^3, 6^, no epidemiological studies have quantified or characterized the correlation between CL and skin cancer.

### Limitations

We did not systematically search grey literature, yet we argue that it would have provided few or no additional relevant references. Even if there were some references, the overall conclusion would hold: the association between CL and skin cancer has been seriously understudied.

## Conclusions

Evidence based on clinical reports ^3, 6^, along with solid physiopathology pathways ( Table 2) ^4, 5^, supports the argument that the association of interest is not random or due to “bad luck”. Nonetheless, and even though both CL and skin cancer impose a non-negligible health burden with larger impact in LMICs, the epidemiological work on this association appears to be null. Research is much needed to verify (or disprove) the available evidence, thereby enabling the development of pragmatic tools to help and guide early diagnosis or prevention of skin cancer among survivors of CL. 

**Table 2.** Selected physiopathology pathways linking cutaneous leishmaniasis (CL) as a potential risk factor for skin cancer.

Reference	Proposed physiopathology pathway(s)
Schwing A, Pomares C, Majoor A, Boyer L, Marty P, Michel G. Leishmania infection: Misdiagnosis as cancer and tumor-promoting potential. Acta Trop. 2018. pii: S0001-706X(18)31322-6.	Among others, this work recaps some proposed pathways: i) through disturbing the activation and functioning of inflammation cells (e.g., macrophages and dendritic cells), Leishmaniasis could be responsible for chronic inflammation, a risk factor for neoplasms; ii) Leishmaniasis may promote a micro-environment rich in Th2 response which, along with the chronic inflammation, may initiate the transition towards cancer.
Kopterides P, Mourtzoukou EG, Skopelitis E, Tsavaris N, Falagas ME. Aspects of the association between leishmaniasis and malignant disorders. Trans R Soc Trop Med Hyg. 2007;101(12):1181-9.	Basal cell carcinoma arising in scars of old CL lesions (i.e., dysplastic changes). Scars and sun exposure are risk factors for malignancies.
Kargi E, Güngör E, Aslan G, Erdogan B. Epidermoid carcinoma in cutaneous leishmaniasis scar. Ann Plast Surg. 2001;46(6):657-8.	As part of a case report summarizes previous findings suggesting that: i) the development of neoplastic lesions where there had been a CL scar known consequence; ii) the development of tumours on cutaneous scars is not new; and iii) basal cell carcinoma could be a consequence of CL lesions (from Suster *et al*., 1988).
Suster S, Ronnen M. Basal cell carcinoma arising in a Leishmania scar. Int J Dermatol. 1988;27(3):175-6.	They recap an old theory suggesting that the effect of ultraviolet radiation and other environmental carcinogens may be exacerbated in tissues with a reduced vascularity and atrophy of adnexal structures, as it is the case when there are scarring processes.
	A consistent consequence seems to be that sun exposure, i.e., ultraviolet radiation, could have stronger negative on CL lesions than on lesions-free skin. Noteworthy, CL patients are mostly from rural areas or fieldworkers whom are constantly exposed to sun light. The pathophysiological evidence may suggest that these groups are of particular relevance for this association and deserves further research.

## Data availability

### Underlying data

All data underlying the results are available as part of the article and no additional source data are required.

### Reporting guidelines

Figshare: PRISMA checklist for article ‘Is there an association between cutaneous Leishmaniasis and skin cancer? A systematic review’. https://doi.org/10.6084/m9.figshare.8870690.v1 ^7^.

The completed PRISMA checklist is available under the terms of the Creative Commons Attribution 4.0 International license (CC-BY 4.0).
