# Supplementary material for: Is there an association between cutaneous leishmaniasis and skin cancer? A systematic review
Source: Wellcome Open Res. 2019 Jul 23;4:110. [Version 1] doi: 10.12688/wellcomeopenres.15367.1 (PMC6719669; doi:10.12688/wellcomeopenres.15367.1)
Supplement: Peer review history

# Peer review: Reviewer response for version 1

**DOI:** 10.21956/wellcomeopenres.16787.r36151

**Published:** 2019-08-28

**Authors:** Temmy Sunyoto

**Affiliations:** 1 Department of Public Health, Institute of Tropical Medicine Antwerp, Antwerp, Belgium; 2 Unit of Epidemiology and Control of Neglected Tropical Disease, Institute of Tropical Medicine Antwerp, Antwerp, Belgium

Thank you for the opportunity given to review this paper.

 In this succinct text, the authors carried out a systematic review aiming to summarise available evidence on the epidemiological association between cutaneous leishmaniasis and skin cancer. The title is clear and using a question format reflects the research question and the method that was applied to answer it. The topic is also of interest, especially when relations, if any, between communicable/infectious diseases and non-communicable diseases, are rarely explored. 

 Reading the text truly feels as a refresher, compared to reading manuscripts with lengthy and too many details. In general, the authors have succeeded in stripping the non-essential elements and remain to the point. The procedure for systematic review was explained clearly and adhered to the standard. From the screening and identification, finally, no record/study was found to be eligible. Therefore the authors point to the inexistence of evidence between CL and skin cancer, in the published literature. 

 Of note, I would like to express my appreciation for this 'negative' result publication. More often than not, journals are reluctant to publish manuscripts with non-positive or non-significant results. The fact that this systematic review yielded no suitable paper would actually help to design further research questions or designs to answer this knowledge gap. In fact, this paper can serve as an example of justification on why studying this topic is important - it shows that evidence is very limited. For any epidemiological study, or maybe all studies, this literature review step is crucial and I commend again the authors and the journal for the work done. 

 Now more specifically, I do have some comments that I hope will be useful for the authors:

- The introduction, albeit concise, lack a bit the depth of the spectrum of cutaneous leishmaniasis, or even the critical detail of CL being a parasitic protozoan disease. CL can manifest locally, as a single lesion, or disseminated and complicated, or even the mucocutaneous forms. From Table 1 (search terms) we could glean that the authors did cover all forms of CL during the search, but for readers not too familiar with CL it is perhaps useful to add one or two sentences that reflect the complexity of CL.
- Related to that, even though no record is included, I wonder if in the pathophysiological pathways linking CL and skin cancer (Table 2) whether the different clinical manifestations could play a role. Furthermore, at least the existing evidence can be elaborated as per the origin of the case reports/suspicion (e.g Old World vs New World).
- In the Methods section, in the first paragraph 'Protocol and Registration' - the last sentence about PRISMA guidelines is citing the figure of the paper itself. Although this is not strictly incorrect, especially with FigShare citeable characteristics, another reference (e.g Mohler, Cochrane etc) is also necessary. 
- Did the authors also screen the reference list from the full-text articles or contact research groups working on the issue? To be fair, indeed this may not yield much addition, but for a systematic review I find it to be important to ensure that all information sources have been exhausted. 
- In results, although the 6 full text articles were considered non-eligible to be included in the synthesis, it will be informative to understand more about them: are they case reports reporting a single patient? I know that as they are not filling inclusion criteria there is no need to elaborate, but as a curious reader, I am wondering why some are included in Table 2 (Kopterides, Schwing) and some are not. 
- In terms of discussion and conclusion, the straightforward conclusion that CL and skin cancer association is understudied is valid. However, I think it will be worthwhile to expand a bit more on the recommendations for future research, and not just broadly in terms of 'enabling the development of pragmatic tools to help and guide early diagnosis or prevention of skin cancer among survivors of CL'. As the authors have stated, the double burden of diseases is important in LMIC, so this paper should also serve as a call to increase awareness in the context where the diagnosis and treatment are limited for patients with either condition.

Looking forward to seeing the next version and again thank you to the authors' team for the attention to the neglected yet intriguing aspect of CL.

I confirm that I have read this submission and believe that I have an appropriate level of expertise to confirm that it is of an acceptable scientific standard.

# Peer review: Reviewer response for version 1

**DOI:** 10.21956/wellcomeopenres.16787.r36033

**Published:** 2019-08-02

**Authors:** Ibrahim Khalifeh

**Affiliations:** 1 Department of Pathology and Laboratory Medicine, American University of Beirut Medical Center, Beirut, Lebanon

The manuscript entitled “Is there an association between cutaneous leishmaniasis and skin cancer? A systematic review” studied the potential correlation between cutaneous leishmaniasis (CL) and skin cancer. The aim of this review is to study the link between these two diseases, which has not been well elucidated before. The manuscript is clear, concise and well written.

 In the Introduction, authors covered both diseases succinctly, and enlighten the need of new studies correlating them.

 The results covered around 1,400 studies without a clear identification of an association between CL and skin cancer. Authors summarized reports with relevant evidence about the correlation between these two diseases. Thus, presentation of a high number of studies along with no studies providing a clear association between CL and skin cancer is worth investigating, and sheds light on the necessity of new approaches for a better guidance and diagnosis.

I confirm that I have read this submission and believe that I have an appropriate level of expertise to confirm that it is of an acceptable scientific standard.
